# Exopolysaccharide composition and size in *Sulfolobus acidocaldarius* biofilms

**DOI:** 10.3389/fmicb.2022.982745

**Published:** 2022-09-26

**Authors:** Laura Kuschmierz, Martin Meyer, Christopher Bräsen, Jost Wingender, Oliver J. Schmitz, Bettina Siebers

**Affiliations:** ^1^Molecular Enzyme Technology and Biochemistry, Environmental Microbiology and Biotechnology, Centre for Water and Environmental Research, University of Duisburg-Essen, Essen, Germany; ^2^Applied Analytical Chemistry, University of Duisburg-Essen, Essen, Germany; ^3^Teaching and Research Center for Separation, University of Duisburg-Essen, Essen, Germany; ^4^Aquatic Microbiology, Environmental Microbiology and Biotechnology, Centre for Water and Environmental Research, University of Duisburg-Essen, Essen, Germany

**Keywords:** *Sulfolobus acidocaldarius*, Archaea, biofilm, extracellular polymeric substances, exopolysaccharide, reversed-phase liquid chromatography, size exclusion chromatography

## Abstract

Extracellular polymeric substances (EPS) comprise mainly carbohydrates, proteins and extracellular DNA (eDNA) in biofilms formed by the thermoacidophilic Crenarchaeon *Sulfolobus acidocaldarius*. However, detailed information on the carbohydrates in the *S. acidocaldarius* biofilm EPS, i.e., the exopolysaccharides (PS), in terms of identity, composition and size were missing. In this study, a set of methods was developed and applied to study the PS in *S. acidocaldarius* biofilms. It was initially shown that addition of sugars, most significantly of glucose, to the basal N-Z-amine-based growth medium enhanced biofilm formation. For the generation of sufficient amounts of biomass suitable for chemical analyses, biofilm growth was established and optimized on the surface of membrane filters. EPS were isolated and the contents of carbohydrates, proteins and eDNA were determined. PS purification was achieved by enzymatic digestion of other EPS components (nucleic acids and proteins). After trifluoroacetic acid-mediated hydrolysis of the PS fraction, the monosaccharide composition was analyzed by reversed-phase liquid chromatography (RP-LC) coupled to mass spectrometry (MS). Main sugar constituents detected were mannose, glucose and ribose, as well as minor proportions of rhamnose, *N*-acetylglucosamine, glucosamine and galactosamine. Size exclusion chromatography (SEC) revealed the presence of one single PS fraction with a molecular mass of 4-9 × 10^4^ Da. This study provides detailed information on the PS composition and size of *S. acidocaldarius* MW001 biofilms and methodological tools for future studies on PS biosynthesis and secretion.

## Introduction

In nature, the majority of microorganisms is estimated to exist in a “continuum of aggregations” as flocs, sludges, or films, called biofilms (Moshynets and Spiers, 2016; Flemming and Wuertz, 2019). They are defined as aggregates of microorganisms that usually accumulate at interfaces or aggregate with themselves and that are encased in a self-produced matrix of hydrated extracellular polymeric substances (EPS) (Flemming and Wingender, 2010). EPS comprise biopolymers such as exopolysaccharides (PS), proteins, glycoproteins, nucleic acids, and lipids.

In total, the matrix of EPS builds the scaffold for the biofilm structure and enables surface adhesion, structural and mechanical stability and cohesion as well as the close proximity of cells. As a protecting “house of biofilm cells” (Flemming et al., 2007) it increases cells’ tolerance to antimicrobials and other environmental stresses, e.g., pH or temperature shifts, and the tolerance to organic solvents (Koerdt et al., 2010; Benninghoff et al., 2021). Each of the EPS components contributes to the functionality and stability of the matrix, and each of them is assigned to a set of distinct functions. PS are ubiquitous in the biofilm matrix. Bacterial PS have been reported to be homo- and more frequently heteropolysaccharides with molecular mass ranging from approximately 2.0 × 10^4^ to 1.9 × 10^7^ Da (Mata et al., 2006, 2008; Sutherland, 2017; Wang et al., 2019). PS display a high diversity in their composition of neutral and charged carbohydrate residues which can contain organic and inorganic substituents (Sutherland, 2017). Multiple functions of PS include adhesion of cells to interfaces (initial colonization and long-term attachment of biofilms to surfaces), cell aggregation, cohesion of biofilms by forming a hydrated polymer network determining structure and architecture of biofilms, and protection from diverse external stresses such as desiccation, antimicrobial compounds, host immune defenses or predation by protozoa (Flemming and Wingender, 2010; Limoli et al., 2015). EPS proteins share some functions with PS, and additionally exhibit unique properties, for instance, their enzymatic activities that enable modification of EPS and digestion of extracellular macromolecules for nutrient acquisition (Flemming and Wingender, 2010). eDNA supports adhesion, aggregation as well as cohesion (Whitchurch et al., 2002) and enables horizontal gene transfer (Flemming, 2016). Biofilm structure, integrity and biology depend not only on EPS composition, but also on functional interactions between PS, proteins and eDNA, sometimes in conjunction with other substances (e.g., signaling molecules, mutivalent inorganic ions) in the biofilm matrix. Functional interactions result, for example, in promoting cell aggregation, enhancing stability of the EPS matrix, or retaining and stabilizing extracellular matrix enzymes (Flemming and Wingender, 2010; Tielen et al., 2013; Sutherland, 2017; Reichhardt et al., 2020). Functional interactions essentially contribute to the generation of emergent properties of biofilms (Flemming et al., 2016).

Information on the molecular composition and chemical as well as physical properties of EPS is limited and mostly reported for bacterial monospecies biofilms of clinical relevance (Seviour et al., 2019). Here, the variety of EPS biopolymers and the difficulty in extracting and analyzing them, display challenging hurdles to overcome. Further, both the ratios of EPS polymers as well as their composition change depending on environmental conditions. Cells actively modify the excreted EPS of their matrix along the biofilm life cycle, remodeling their close environment as a function of time and environmental conditions (Flemming, 2011; Seviour et al., 2019).

While methods for high-throughput protein identification are well established, PS analysis methods lack mostly due to the high structural diversity of carbohydrates (Seviour et al., 2019). The complexity of PS analyses is increased by the fact that one strain may produce diverse PS molecules (Ryder et al., 2007). Initially, the EPS matrix composition is typically determined by biochemical, mostly colorimetric, assays that enable the calculation of component sum parameters and their ratios (Felz et al., 2019). The in-depth analysis of EPS components might require complex sample preparation and a significant quantity of a “sufficiently pure compound” (Seviour et al., 2019). For PS size determination gel permeation chromatography/size exclusion chromatography (GPC/SEC) (Simon et al., 2009; Ras et al., 2011) is typically applied. Determination of the composition requires cleavage of the PS into its monomers, which is usually accomplished by acid hydrolysis. A wide range of methods has been published, such as high-performance liquid chromatography (HPLC) (Qamar et al., 2021), high-performance anion-exchange chromatography (HPAEC) (Zhang et al., 2019), capillary electrophoresis (CE) (Gao et al., 2010), and GC after derivatization (Parra-Riofrío et al., 2020).

Compared to bacterial biofilms, substantially less information is available on the formation, regulation, EPS synthesis as well as composition of archaeal biofilms. Archaea were shown to be ubiquitous in nature and are reported to form biofilms as their predominant lifestyle (van Wolferen et al., 2018). Most cultivable archaeal species to date are extremophilic organisms that are adapted to extreme environmental conditions such as extremes of temperature, pH, salt, or combinations thereof. Aggregates of methanogenic archaea, *Halobacterium*, *Haloferax* and members of the Sulfolobales display the best-studied archaeal biofilms (van Wolferen et al., 2018). *Sulfolobus acidocaldarius* is adapted to both high temperature (75–80°C) and low pH values (pH 2–3), and forms complex, dense carpet-like biofilms with tower like structures (Koerdt et al., 2010). The extracellular proteome of *S. acidocaldarius* DSM639 biofilms was proven to comprise numerous enzyme classes, including proteases, lipases, esterases, phosphatases and glucosidases (Jachlewski et al., 2015). In contrast, limited information is available on *S. acidocaldarius* PS. Extracellular carbohydrates were visualized by confocal laser scanning microscopy (Koerdt et al., 2010; Benninghoff et al., 2021). Here, interactions of fluorophore-labeled lectins to PS structures indicated the presence of glucose and/or mannose, as well as lower amounts of galactose and/or *N*-acetyl-D-galactosamine residues. However, the complete and precise monomeric composition and size of the *S. acidocaldarius* PS were not studied so far by chemical analytical techniques.

This study fills the gap of knowledge on *S. acidocaldarius* EPS matrix composition with a special focus on the size of excreted PS as well as the identity of their constituent monosaccharides as a basis for the investigation of PS function for the development and integrity of archaeal biofilms.

## Materials and methods

### Strain and growth conditions

*Sulfolobus acidocaldarius* MW001 was used to generate biofilms. The strain is a deletion mutant of *S. acidocaldarius* DSM639, lacking 322 bp of *pyrE* (Wagner et al., 2012), enabling genetic selection and engineering via uracil auxotrophy. The strain was cultivated aerobically in basal Brock medium (Brock et al., 1972), supplemented with 0.1% (*w/v*) N-Z-amine (NZA; casein enzymatic hydrolysate N-Z-Amine^®^ AS, Sigma-Aldrich, Darmstadt, Germany), 10 μg/mL uracil (min. 99%, Sigma-Aldrich) and different additional growth substrates at 76°C and pH 3.0 (adjusted with 50% (*v/v*) sulfuric acid): (i) 0.2% (*w/v*) D(+)-glucose (p.a., ACS, Carl Roth, Karlsruhe, Germany), (ii) 0.2% (*w/v*) D-xylose (min. 99%, Carl Roth), and (iii) 0.2% (*w/v*) D(+)-maltose (biochemical grade, Gerbu, Heidelberg, Germany). Cell growth was monitored by turbidity measurements at 600 nm.

### Microtiter plate assay

To evaluate biofilm formation of *S. acidocaldarius* in dependency of different growth substrates, cells were grown in 24-well microtiter plates (MTP) (Cell+, flat bottom, polystyrene, Sarstedt, Nümbrecht, Germany). Each well was filled with 2 mL culture volume of an optical density at 600 nm (OD_600*nm*_) of 0.01. Plates were sealed with aluminum foil (aluminum sealing tape, pierceable, Greiner Bio-One, Frickenhausen, Germany) to limit medium evaporation. The foil was pierced once with a sterile needle for each well to ensure oxygen supply. Plates were cultivated for 1–5 days under static conditions at 76°C. After incubation, one set of MTP was used to determine total turbidity (OD_600*nm*_) values of biofilm and planktonic cells after extensive mixing of cultures (Biophotometer plus, Eppendorf, Hamburg, Germany). Another set of MTP was used to measure OD_600*nm*_ values of planktonic cells only. The remaining biofilm biomass in each well was quantified by crystal violet (CV) staining. Briefly, wells were washed once with Brock medium (pH 3.0) to remove planktonic cells. Then, 2.5 mL of 0.1% (*w/v*) crystal violet solution were added into each cavity, incubated for 30 min at RT and washed five times with 2.5 mL dH_2_O. After air-drying, each well was filled with 2 mL of 95% (*v/v*) ethanol and the plates were incubated for 30 min at RT to release the dye from biofilm into solution. The absorbance of crystal violet was measured at 595 nm (Biophotometer plus, Eppendorf). Medium samples served as negative controls and CV values of medium controls were subtracted from CV values of *S. acidocaldarius* biofilms. Ratios of biofilm vs. planktonic cells were calculated by dividing the absorbance of crystal violet (OD_595*nm*_) by growth of planktonic cells (OD_600*nm*_ values) (Koerdt et al., 2010). All experiments were performed in quadruplicates.

### Determination of total cell counts

To determine total cell counts of *S. acidocaldarius* MW001 cultures and cell suspensions the correlation between total cell counts and OD_600*nm*_ values was determined using the improved Neubauer counting chamber (0.0025 mm^2^, 0.020 mm depth, Brand, Wertheim, Germany) by phase-contrast microscopy (Leica DMLS bench microscope, Leica, Wetzlar, Germany). *S. acidocaldarius* MW001 liquid cultures were grown in Brock medium, pH 3.0, supplemented with 0.1% (*w/v*) NZA, 0.2% (*w/v*) D-glucose, 10 μg/mL uracil at 75°C and 180 rpm. Cultures were 10- to 40-fold diluted with 70% (*v/v*) sterile glycerol to enable a reliable counting of the cells under the microscope. For each dilution, cell numbers in six different squares were counted. Cell counts and corresponding OD_600*nm*_ values were plotted.

### Generation of *Sulfolobus acidocaldarius* MW001 biofilms: Membrane filtration and cultivation

To generate *S. acidocaldarius* biofilms, cells were pregrown for three passages in liquid Brock medium containing 0.1% (*w/v*) NZA, 10 μg/mL uracil and optionally 0.2% (*w/v*) D-glucose, D-xylose, or maltose, pH 3.0, at 180 rpm and 75°C. Cells from mid-exponential growth phase (OD_600*nm*_ approx. 0.6-0.8) were then diluted with Brock medium yielding a total cell number of 5.45 × 10^4^ cells/mL. Eleven mL of this culture dilution were filtered through filter membranes composed of different materials (summarized in Supplementary Table 1) using a stainless steel filtration system (Sartorius AG, Goettingen, Germany) connected to a vacuum pump (Liquiport, KNF Neuberger, Trenton, NJ, USA). Finally, highest biofilm yields were obtained with hydrophilic polytetrafluoroethylene (PTFE) membranes (PTFE polymer membrane bound to a high density polyethylene support, Omnipore membrane, 0.45 μm pore size, Ref.no. JHWP04700, Merck-Millipore, Darmstadt, Germany), which were further on used as standard membrane for biofilm cultivations. After filtration, the membranes were placed on the surface of 30 mL liquid Brock medium in Petri dishes (92 mm × 16 mm, polystyrene, without cams, Sarstedt) or on top of solidified Brock medium plates in initial experiments (Supplementary Table 1; 0.6% (*w/v*) gellan gum, Serva Electrophoresis, Heidelberg, Germany) supplemented with the different sugars as mentioned above. Petri dishes were statically incubated at 76°C for 4 days. Biofilms formed on the surface of membranes. For further EPS/PS extraction and analyses, the biofilm mass from 10 membranes was harvested and pooled. Negative controls for RP-LC-MS and SEC analyses were generated by the filtration of medium and subsequent incubation.

### Isolation of extracellular polymeric substances

After cultivation, biofilms were harvested from membranes using a cell scraper (non-cytotoxic, non-pyrogenic, sterile, blade width: 1.70 cm, blade material: thermoplastic elastomers, Sarstedt). EPS extraction was performed using a cation exchange resin (CER, Dowex^®^ Marathon^®^ C sodium form, Sigma-Aldrich; according to Jachlewski et al., 2015). Briefly, biofilm cells were suspended in phosphate buffer (10 mg biofilm wet weight/mL buffer; 2 mM Na_3_PO_4_ × 12 H_2_O, 4 mM NaH_2_PO_4_ x 1 H_2_O, 9 mM NaCl, 1 mM KCl, pH 7.0). Ten milliliter biofilm suspension were treated with 2 g of hydrated CER for 20 min under shaking conditions. Centrifugation and sterile filtration of the supernatant (polyvinylidene fluoride (PVDF) filter Rotilabo, pore size 0.22 μm, Carl Roth) yielded the fraction of total extracellular material (TEM), containing cell-free low and high molecular weight compounds. To obtain the fraction of high molecular weight compounds only (EPS), the TEM sample was dialyzed against Milli-Q water using a membrane tube with a molecular weight cut-off (MWCO) of 3.5 kDa (Spectra/Por3, Standard RC Tubing Dialysis Membrane, ø11.5 mm, Spectrum Laboratories, Waltham, MA, USA) (Jachlewski et al., 2015). As negative controls for RP-LC-MS and SEC analyses medium samples from membrane filtration were used (see section “Generation of *Sulfolobus acidocaldarius* MW001 biofilms: Membrane filtration and cultivation”). Despite the absence of biofilms, the protocol for EPS isolation was followed as described for biofilm samples.

### Quantification of EPS components

Carbohydrates and proteins were quantified in biofilm suspensions, TEM and EPS samples as described by Jachlewski et al. (2015). Proteins were quantified according to a modified Lowry assay (Peterson, 1977) using bovine serum albumin as standard (Serva Electrophoresis; calibration ranging from 0 to 100 μg/mL) (Wingender et al., 2001). Carbohydrates were quantified using the phenol/sulphuric acid method (Dubois et al., 1956) with D-glucose as standard (p.a., ACS, Carl Roth; calibration ranging from 0 to 200 μg/mL). eDNA was quantified using the Qubit™ dsDNA HS Assay Kit and the Qubit™ 3 Fluorometer (Invitrogen, Thermo Fisher Scientific, Waltham, Massachusetts, USA).

### Spotting plates

Biofilms were grown on membrane filters for 4 days at 76°C. Then, biofilms were harvested from the filter using a cell scraper (Sarstedt) and resuspended in phosphate buffer as described in section “Isolation of extracellular polymeric substances”. Biofilm suspensions were diluted in a 10-fold dilution series (10^–1^–10^–7^) using Brock medium, pH 5.0–5.5. Ten microliter of biofilm suspensions and dilutions were spotted on Brock-gellan gum plates containing 0.6% (*w/v*) gellan gum (Gelzan, Sigma-Aldrich), 0.1% (*w/v*) NZA, 0.2% (*w/v*) D-glucose and 10 μg/mL uracil. Plates were incubated for 5 days at 76°C. Culturability was documented by camera.

### Acid hydrolysis of exopolysaccharides

The hydrolysis method was adapted and modified from de Swaaf et al. (2001). Prior to hydrolysis, 1 mL of extracted EPS solution was evaporated to dryness in a vacuum centrifuge (Concentrator Plus, Eppendorf) at 45°C in vacuum. The dried EPS were dissolved in 150 μL of 2 M trifluoroacetic acid (TFA) (HPLC grade, AppliChem) and the solution was incubated in a heating cabinet at 95°C for 3 h. Afterward, samples were evaporated to dryness using a parallel evaporation concentrator (Shaker: Syncore; Pump: Vac V-500; Controller: Vacuum Controller V-800; Büchi Labortechnik, Essen, Germany) at 50°C, 100 mbar and 200 rpm. For neutralization and washing, 100 μL of 1 M NH_4_OH solution (Suprapur, Merck) were added and after vortexing for 10 s, the solution was evaporated to dryness as described above. Finally, samples were dissolved in 500 μL of ultrapure water and the solutions were vortexed for 10 s. Samples that were not directly analyzed, were stored at -20°C until further use.

### Glycan labeling and fluorescence-assisted carbohydrate electrophoresis

Glycans were labeled at their reducing ends via reductive amination (Ruhaak et al., 2010) using 2-aminonaphthalene trisulfonic acid (ANTS; Invitrogen, Thermo Fisher Scientific). Solutions of 25 mM ANTS in Milli-Q water/acetic acid (17:3 (*v/v*); 99.8-100%, Bernd Kraft, Duisburg, Germany) and 1 M sodium cyanoborohydride (Sigma-Aldrich) in dimethyl sulfoxide (DMSO; 99.7%, Acros Organics, NJ, USA) were prepared. As glycan standards 0.15% (*w/v*) D(+)-glucose (p.a., ACS, Carl Roth), 0.15% (*w/v*) D(+)-maltose (biochemical grade, Gerbu) and 20% (*w/v*) dextrin (pure, Carl Roth) solutions were used. EPS and acid hydrolyzed EPS samples were concentrated 30-fold (SpeedVac, Concentrator Plus, Eppendorf). Twenty-five microliter of samples/glycan standard solutions or Milli-Q water (negative control) were mixed with 90 μL of 25 mM ANTS solution and 100 μL of 1 M sodium cyanoborohydride. Reaction mixtures were vortexed and incubated in the dark at 37°C overnight. ANTS-labeled glycans were visualized using fluorescence-assisted carbohydrate electrophoresis (FACE). Thirty% FACE gels were poured using Rotiphorese acrylamide/bisacrylamide solution 40 (37,5:1; Carl Roth), 10x Tris-acetate-EDTA buffer, 10% (*w/v*) ammonium peroxodisulfate (Carl Roth), TEMED (Carl Roth), and dH_2_O. DMSO was removed from labeling reaction mixtures using the SpeedVac (Concentrator Plus, Eppendorf) at 60°C for 4 h. The sample volumes were adjusted to 100 μL with Milli-Q water, then 10 μL of labeled sample and 90 μL of 50% (*v/v*) glycerol solution were mixed. Five microliter of each sample were applied to FACE. For electrophoresis (Mini-PROTEAN Tetra cell system, Bio-Rad) a constant electrical voltage of 300 V was applied for 30 min in TAE buffer. Based on the negative charge of ANTS, labeled glycans are separated according to their molecular size by electrophoresis. ANTS-mediated UV absorbance was visualized using the Molecular Imager^®^ Gel Doc™ XR System (Bio-Rad).

### Derivatization of monosaccharides

Prior to analysis by liquid chromatography, the monosaccharides from references and hydrolyzed PS samples were derivatized with 1-phenyl-3-methyl-5-pyrazolone (PMP). The derivatization method was adapted and modified from the ones originally reported by Honda et al. (1989) and Rühmann et al. (2015). As derivatization agent, a 0.1 M PMP solution (≥99%, Sigma-Aldrich) in methanol (LC-MS grade, VWR) and 0.4% ammonium hydroxide (Suprapur, Merck) were mixed in a volume ratio of 2:1. Twenty-five microliter of sample were mixed with 75 μL of derivatization agent, vortexed and centrifuged at 268 × *g* for 2 min at room temperature (MiniSpin Plus, Eppendorf). After that, the samples were incubated at 70°C for 100 min in a heating cabinet. Then, 25 μL of 0.5 M acetic acid (glacial, VWR) and 875 μL of ultrapure water were added. Each sample was filtered (pore size 0.2 μM, cellulose, CS—Chromatographie Service, Langerwehe, Germany) and transferred to LC vials. Samples which were not directly analyzed, were stored at -20°C until further use. For more details see Meyer et al. (2022).

### Analysis of monosaccharide composition

The monosaccharide composition analysis in EPS from *S. acidocaldarius* biofilms has been carried out using reversed phase liquid chromatography (RP-LC) coupled to triple quadrupole (TQ) mass spectrometry (MS) in multiple reaction monitoring (MRM) mode. An Agilent 1290 Infinity II LC system was coupled to an Agilent Triple Quadrupole mass spectrometer. The LC/TQ system comprised a 1290 high speed pump (G7120A, Agilent, Santa Clara, CA, USA), a 1290 Vialsampler (G7167B, Agilent), a 1290 multicolumn thermostat (MCT G7116B, Agilent) and a LC/TQ (G6470B, Agilent). A Kinetex C18 (100 × 2.1 mm, 1.7 μm core shell particles) (Phenomenex, Torrance, USA) column was used and the separation was carried out at 50°C. The mobile phase contained a 5 mM ammonium acetate buffer, pH 5.6 (LiChropur LC-MS, Merck) with 15% (*v/v*) acetonitrile (mobile phase A) and pure acetonitrile (mobile phase B) (LC-MS grade, VWR). The optimized gradient time was 15 min at a constant flow rate of 0.5 mL/min. The gradient started at 0% B, to 1% B in 2 min, increased to 5% B in 5 min and held for 2 min; to 18% B in 1 min, further increased to 40% B in 0.3 min and held for 2 min. Prior to the next injection the column was equilibrated at initial conditions for 2.7 min.

Mass spectrometry was performed using an electrospray ionization source (ESI) operating in positive ion mode, with a 2.5 min cut off to remove the early eluting excess of PMP. Nitrogen was utilized as sheath gas, drying gas and collision gas. The sheath and drying gas flow rates were set to 8 and 6 L/min at 325°C, respectively. The nebulizer was set at 40 psi and the capillary voltage and nozzle voltage were set at 4000 and 500 V, respectively. The fragmentor and cell acceleration voltage (CAV) were set to 165 and 9 V, respectively. The acquisition mode was performed on MS/MS in MRM with transitions of [M-OH + 2PMP]^+^ to [PMP + H]^+^ at optimized collision energies (20 and 30 V) for each monosaccharide using a dwell time of 100 ms. The LC and MS system were controlled by the software Agilent Mass Hunter Workstation Data Acquisition (Version C.01.00), data evaluation was carried out using Agilent Mass Hunter Qualitative Analysis Navigator (Version B.08.00). A mixture of 15 separable monosaccharides was prepared and limit of detection (LOD) and quantification (LOQ) were determined by analyzing dilutions of this mixture in fifteen calibration levels as triplicates (100, 80, 60, 40, 20, 10, 5, 2, 1, 0.5, 0.2, 0.1, 0.05, 0.02, and 0.01 μmol/L for each monosaccharide). LOD of the RP-LC-MS method was calculated with minimum 3× and LOQ with minimum 9× signal-to-noise ratio. LODs and LOQs (as μg/L) for the separated monosaccharides, and additionally, coefficient of determination (r^2^), retention time (rt) and mass as derivatized compound [M-OH + 2PMP], are given in Supplementary Table 3. EPS samples were analyzed in triplicates in blocks by RP-LC. Between the blocks, one blank was injected and additionally, before each injection, the injection needle was washed thoroughly, to avoid carryover and cross-contamination of analytes from the different samples. For the correction of potential contamination effects and the prevention of a monomer overestimation in EPS samples, negative control samples were treated with hydrolysis and derivatization and were analyzed. Detected monosaccharides in negative controls were quantified and subtracted from quantification results of real biofilm EPS samples, to rule out false-positive assignment of monosaccharides not originating from *S. acidocaldarius* biofilms.

### RNaseA, DNaseI, and proteinase K treatment of TEM samples

To reduce the amounts of non-carbohydrate molecules in EPS samples other EPS components, namely extracellular nucleic acids and proteins, were digested. TEM fractions of *S. acidocaldarius* MW001 biofilms were treated with RNaseA to digest extracellular RNA (eRNA). Ten μg/mL RNaseA (Roche Diagnostics, Mannheim, Germany; stock solution of 10 mg/mL in 10 mM TrisHCl, 15 mM NaCl, pH 7.5) were added to TEM samples and incubated at 37°C overnight. This was followed by either further enzymatic treatments or by the dialysis of RNaseA treated TEM sample to generate the fraction of EPS molecules after RNaseA treatment (MWCO 3.5 kDa or 12–14 kDa; Spectrum Laboratories). DNaseI treatment was used to digest eDNA in the TEM fraction of *S. acidocaldarius* MW001 biofilms. Here, 10 μg/mL DNaseI (AppliChem, Darmstadt, Germany; stock solution of 10 mg/mL in 20 mM TrisHCl, 1 mM MgCl_2_, 50% (*v/v*) glycerol, pH 7.5) and 10 mM MgCl_2_ were added to TEM samples and incubated at 37°C for 1 h. To remove co-isolated (possibly glycosylated) EPS proteins as well as RNaseA and DNaseI, TEM samples were treated with proteinase K. About 100 μg/mL proteinase K (GeneOn, Ludwigshafen, Germany; 20 mg/mL) and 1 mM CaCl_2_ were added to TEM fractions of *S. acidocaldarius* biofilms. Incubation was performed at 37°C overnight. The enzymatically treated TEM sample was dialyzed against Milli-Q water using a MWCO of 3.5 kDa.

### SDS-PAGE analyses of EPS proteins

To visualize EPS proteins EPS fractions were analyzed by SDS-PAGE. Samples treated with and without proteinase K were concentrated 20-fold by trichloroacetic acid (TCA) precipitation. One volume of 20% (*w/v*) TCA (ReagentPlus^®^, 99%, Sigma-Aldrich) was added to two volumes of sample, and the mixtures were incubated on ice for 20 min. After centrifugation (16,363 × *g*, 4°C, 30 min) the supernatant was discarded, the pellets were washed with a full volume of 80% (*v/v*) ice-cold acetone (analytical reagent grade, Fisher Chemical, Fisher Scientific) twice, air-dried for 30 min and resuspended in 50 mM TrisHCl buffer (pH 8.0) to yield 20-fold concentrated protein samples. Two microgram of EPS proteins were loaded on a 12% SDS-PAGE gel. After electrophoresis, EPS proteins were visualized using silver staining (Pierce Silver Stain Kit, Thermo Fisher Scientific) according to the manufacturer’s instructions. Gel documentation was performed using the Molecular Imager^®^ Gel Doc ™ XR System (Bio-Rad, Hercules, CA, USA).

### Size exclusion chromatography

Size exclusion chromatography for determination of molecular weight distribution of PS was performed using a HPLC system. An Agilent 1260 Infinity II SFC/UHPLC hybrid system operating in LC mode was coupled to an Agilent 1290 Infinity II Evaporating Light Scattering Detector (ELSD). The LC-ELSD system comprised a 1260 SFC binary pump (G4782A, Agilent), a 1260 SFC multisampler (G4767A, Agilent), a 1260 MCT (G7116A, Agilent), and a 1290 ELSD (G7102A, Agilent). The used columns were BioSep SEC-s2000 (300 mm × 7.8 mm, 5 μm particle size) and BioSep SEC-s4000 (300 mm × 7.8 mm, 5 μm particle size) (Phenomenex) in series, resulting in size fractionation between 1 and 1,500 kDa according to manufacturer information. Isocratic elution was carried out using water (ultrapure and desalted, resistivity 18.2 MΩ⋅cm, Sartorius) at constant 0.5 mL/min flow rate. Detector parameters were set to 80°C evaporator temperature, 90°C nebulizer temperature and 2.00 SLM (standard liter per minute) nitrogen gas flow rate. Calibration of the SEC columns was performed by injecting ten different size polysaccharide standards (Pullulan Standard Set 350-700,000, analytical standards, Supelco, St. Louis, MO, USA: 342 Da, 1,030 Da, 6,300 Da, 9,800 Da, 22,000 Da, 49,400 Da, 110,000 Da, 201,000 Da, 334,000 Da, 708,000 Da). Calibration resulted in following equation: log(MW) = –0.0004x^3^ + 0.0427x^2^ – 1.4439x + 21.425 with molecular weight (MW) expressed in Da and retention time x in minutes. The LC-ELSD system was controlled by Agilent OpenLAB CDS—Aquisition (Version 2.3), data evaluation was carried out using Agilent OpenLAB Data Analysis (Version 2.3). The calculation of the number of monomeric residues in the PS was performed assuming a linear polymer. The mass of the individual monosaccharide in a glycosidic bond was reduced by 18 atomic units (for water release) and the share of the different monosaccharides in the PS, which was previously determined in the LC-MS analyses, was incorporated in the calculation.

## Results

### *Sulfolobus acidocaldarius* biofilm formation in different growth media

Brock medium (Brock et al., 1972) with 0.1% (*w/v*) N-Z-amine (NZA) was used as complex basal medium for growth. Different sugars were added to this medium and their influence on biofilm formation and quantity was compared in 24-well plates. The sugars used in this study, D-glucose, D-xylose or maltose, had been shown to promote *S. acidocaldarius* growth in liquid cultures in previous studies (Choi et al., 2013; Wagner et al., 2014, 2018). Cultures were inoculated to an OD_600nm_ value of 0.01. Cultivation was performed under static conditions for one to five days at 76°C. Each day (i) total turbidity (OD_600nm_) values of biofilm and planktonic cells, (ii) OD_600nm_ values of planktonic cell suspensions, and (iii) biofilm amounts using crystal violet staining were determined (Supplementary Figure 1). Cultivation with 0.1% (*w/v*) NZA and 0.2% (*w/v*) D-glucose resulted in the highest amounts of firmly attached *S. acidocaldarius* biofilm mass after four days of static cultivation (91 h) (Supplementary Figures 1, 2). The highest ratio of biofilm to planktonic cells was detected by the supplementation of glucose, leading to an at least 1.85-fold higher ratio than observed for the other tested growth media (Supplementary Figure 2D). Hence, this medium composition was used predominantly in subsequent biofilm cultivation and PS analyses.

### Optimization of *Sulfolobus acidocaldarius* biofilm cultivation

**FIGURE 1:**
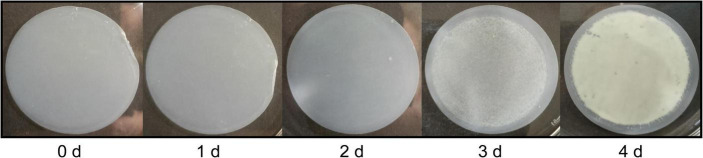
Time-dependent *Sulfolobus acidocaldarius* MW001 biofilm formation on the hydrophilic PTFE membrane. Membranes were inoculated with 6 × 10^5^ cells/membrane, equivalent to a cell density of 3.5 × 10^4^ cells/cm^2^. Cultivation was performed statically floating on liquid Brock medium (pH 3.0) supplemented with 0.1% (*w/v*) NZA, 0.2% (*w/v*) glucose, 10 μg/mL uracil, at 76°C. Biofilm formation was documented by camera every day. After 4 days of cultivation the confluent biofilm was harvested. A cell density of 3.4 × 10^9^ cells/cm^2^ was determined after 4 d (Supplementary Figure 3D).

Up to now, archaeal biofilms were mostly generated in MTPs, dishes or on slides consisting of different types of polymers (e.g., polystyrene) or glass as substratum for biofilm formation (Benninghoff et al., 2017). While the cultivation of biofilms, e.g., in MTPs, is optimal to analyze biofilm properties on a high-throughput scale (see section “*Sulfolobus acidocaldarius* biofilm formation in different growth media”), it is suboptimal to receive high amounts of biofilm mass. The cultivation of *S. acidocaldarius* biofilms on gellan gum-solidified Brock medium plates, as reported by Jachlewski et al. (2015), bears the risk of co-isolation of the gelling agent, an anionic heteropolysaccharide (Jansson and Lindberg, 1983), during EPS extraction. Alternatively, synthetic filtration membranes have been used for the cultivation of biofilms (Vanysacker et al., 2013; Zhang et al., 2014; Waheed et al., 2022). We adapted and optimized this method for the cultivation of *S. acidocaldarius* biofilms. *S. acidocaldarius* MW001 cell suspensions were filtered on a membrane. The membrane was placed on the surface of liquid or gellan gum-solidified Brock medium and incubated statically at 76°C for 4 days. Media components were allowed to pass the pores of membranes and ensured nutrient availability for biofilm growth on top of the membranes. Ten different commercially available membranes were compared regarding their ability to ensure *S. acidocaldarius* biofilm formation. These included hydrophilic and hydrophobic round membranes of different chemical composition and pore sizes of 0.2–0.45 μm (Supplementary Table 1). The three best performing membranes regarding biofilm mass yield (Supplementary Table 1) were chosen for a more detailed comparison of *S. acidocaldarius* biofilm formation on liquid medium, comparing the generated biofilm wet weights per area, total cell counts, culturability and carbohydrate amounts in EPS fractions (Supplementary Figure 3). While heterogeneous biofilms were formed on the polycarbonate track etched filter or on the PTFE filter, a reproducibly homogeneous confluent biofilm was formed on the hydrophilic PTFE membrane (Omnipore, PTFE polymer membrane bound to a high density polyethylene support, 0.45 μm pore size, 47 mm diameter, Merck). Thus, this polymeric membrane was used for biofilm cultivation. Different inocula, in the range of 10^4^–10^8^ cells/membrane, were applied on the membrane and biofilm formation was compared after 4 days of static incubation at 76°C. The filtration of low cell numbers (10^4^) resulted in the formation of macrocolonies after 4 days of cultivation, while the application of high cell numbers (10^7^–10^8^) resulted in the formation of a biofilm that was only loosely attached to the membrane (data not shown). An inoculum of 10^5^–10^6^ cells/membrane led to significant biofilm growth firmly attached to the membrane surface after 4 days of cultivation (Figure 1). In the optimized cultivation protocol, 6 × 10^5^ *S. acidocaldarius* MW001 cells were filtered on the hydrophilic PTFE membrane (Omnipore) and incubated statically floating on liquid Brock medium (0.1% (*w/v*) NZA, 0.2% (*w/v*) glucose, 10 μg/mL uracil) at 76°C for 4 days.

### Quantification of carbohydrates, proteins and eDNA in EPS fractions of *Sulfolobus acidocaldarius* MW001 biofilms

**FIGURE 2:**
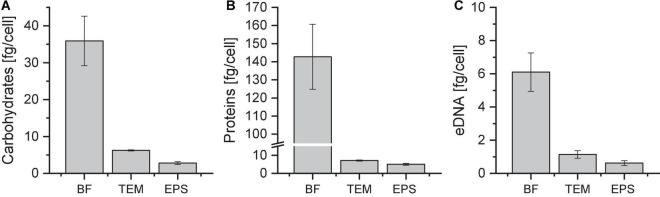
Amounts of carbohydrates **(A)**, proteins **(B)**, and eDNA **(C)** in different *Sulfolobus acidocaldarius* MW001 biofilm fractions. Biofilms were grown on hydrophilic PTFE membranes at 76°C for 4 days (liquid Brock medium, pH 3.0, 0.1% (*w/v*) NZA, 0.2% (*w/v*) glucose, 10 μg/mL uracil). EPS isolation was performed using CER. Carbohydrate, protein, and eDNA concentrations were determined using the phenol/sulfuric acid method, the modified Lowry assay and the Qubit dsDNA assay, respectively. BF, biofilm suspension; TEM, total extracellular material; EPS, extracellular polymeric substances (MWCO 3.5 kDa) (*n* = 3).

Extracellular polymeric substances were isolated from biofilm suspensions with a cation exchange resin (CER) as reported by Jachlewski et al. (2015). After CER treatment, the sterile-filtered extract contained total extracellular material (TEM) of low- and high-molecular-weight compounds. The EPS fraction was obtained by dialysis of the TEM sample, removing low-molecular-weight compounds (MWCO 3.5 kDa). The amounts of carbohydrates, proteins and eDNA per cell were quantified for the biofilm suspension (BF) and the fractions of TEM and EPS of 4 days old biofilms (Figure 2).

With an amount of 5.0 ± 0.5 fg/cell proteins were most abundant in the EPS fraction of *S. acidocaldarius* MW001 biofilms grown on membranes, followed by carbohydrates with an amount of 2.8 ± 0.6 fg/cell and eDNA with 0.6 ± 0.1 fg/cell (Figure 2). The mass ratios of proteins:carbohydrates:eDNA were approx. 8:4:1 under the chosen growth conditions.

### Analysis of exopolysaccharide monomeric composition

To unravel the monosaccharide composition in *S. acidocaldarius* EPS, samples of *S. acidocaldarius* MW001 biofilms were subjected to acid hydrolysis. In preliminary experiments, three different hydrolysis methods were compared, using trifluoroacetic acid (TFA), sulfuric acid or hydrochloric acid, and the corresponding monosaccharide concentration was determined (Supplementary Figure 4A and Supplementary Table 2). Highest carbohydrate concentrations in EPS hydrolysates were observed using TFA for acid hydrolysis. In addition, the method required less extensive neutralization steps and an overall lower sample preparation time and was thus less prone to losses of analytes throughout the procedure. Lower concentrations of individual sugars, as well as overall lower yields for the carbohydrate fraction were determined using sulfuric acid or hydrochloric acid. The success of PS hydrolysis by TFA, meaning the formation of monosaccharides, was also visualized by ANTS carbohydrate labeling and FACE (Supplementary Figure 4B). While the sensitivity of the method did not allow the visualization of PS, TFA hydrolysis led to an increased availability of reducing ends for the labeling reaction and the occurrence of monosaccharide signals in FACE. After 2 h of hydrolysis, a strong monosaccharide signal was present in hydrolyzed EPS samples. The monosaccharide signal intensity did not increase any further after 3 h of hydrolysis (Supplementary Figure 4B).

For the analysis of the monomeric PS composition, reversed-phase liquid chromatography (RP-LC) coupled to mass spectrometry was chosen as an advanced method for analysis. To determine the suitability of the RP-LC method for analysis of the PS composition, single component standards out of a set of 15 monosaccharides, which were selected as they are among the most common naturally occurring monosaccharides, were derivatized with 1-phenyl-3-methyl-5-pyrazolone (PMP) and retention times for each component were assigned. All monosaccharides could be separated either by retention time or mass differences (Supplementary Figure 5; Supplementary Table 3; Meyer et al., 2022).

**FIGURE 3:**
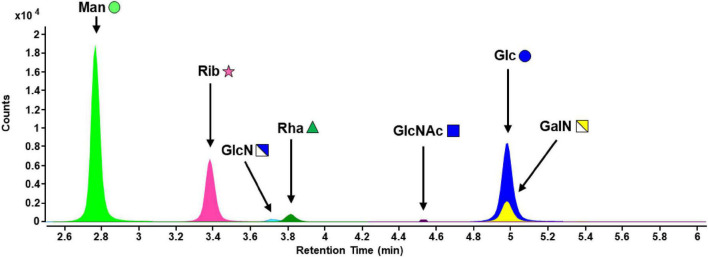
MRM chromatogram of carbohydrates of hydrolyzed EPS from *S acidocaldarius* MW001 biofilms cultivated on hydrophilic PTFE membranes at 76°C for 4 days (liquid Brock medium, pH 3.0, 0.1% (*w/v*) NZA, 0.2% (*w/v*) glucose, 10 μg/mL uracil). Monosaccharide symbols were used according to the glycan nomenclature of NCBI (Man, mannose; Rib, ribose; GlcN, glucosamine; Rha, rhamnose; GlcNAc, *N*-acetylglucosamine; Glc, glucose; GalN, galactosamine). Further information on the analytical performance of the RP-LC-MS method can be found in Supplementary Table 3.

EPS samples from *S. acidocaldarius* MW001 biofilms were processed by TFA hydrolysis and PMP derivatization. Besides FACE analysis, successful hydrolysis and exclusion of remaining poly- and oligosaccharide structures was confirmed by LC-MS in full scan mode with a larger m/z (mass-to-charge ratio) range. No signals were detected above m/z = 600, hence, all PS structures were hydrolyzed to monosaccharides (data not shown). Analysis of the hydrolyzed EPS fraction of biofilm samples resulted in the detection of mannose (Man), ribose (Rib), glucosamine (GlcN), rhamnose (Rha), *N*-acetylglucosamine (GlcNAc), glucose (Glc), and galactosamine (GalN) (Figure 3).

**FIGURE 4:**
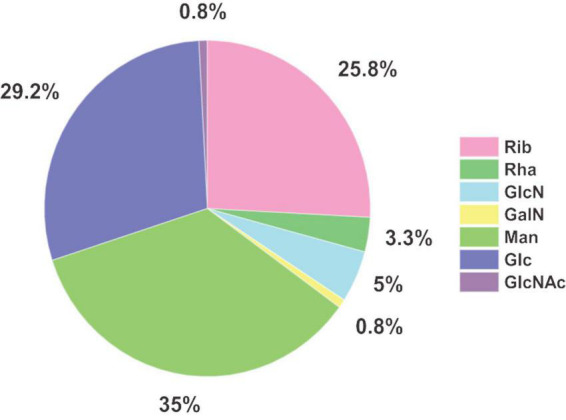
Molar ratios of monosaccharides identified in EPS of *Sulfolobus acidocaldarius* MW001 biofilm cultivated on hydrophilic PTFE membranes at 76°C for 4 days (liquid Brock medium, pH 3.0, 0.1% (*w/v*) NZA, 0.2% (*w/v*) glucose, 10 μg/mL uracil) (*n* = 3). Values and standard deviations of the biological replicates are given in Supplementary Table 5.

Major monosaccharide components detected in EPS were mannose (35%), glucose (29%), and ribose (26%), making up more than 90% of the monosaccharidic composition in terms of molar ratios (Figure 4). Minor components were glucosamine (5%) and rhamnose (3%). Galactosamine and *N*-acetylglucosamine, with a fraction of around 1% each regarding the total concentration, were detected above the limit of quantification (LOQ) and were therefore quantifiable. Due to the fact that acetylated carbohydrate species are usually mostly deacetylated during acid hydrolysis, some share of the galactosamine and glucosamine fraction might originate from *N*-acetylated species such as *N*-acetylgalactosamine and *N*-acetylglucosamine, respectively. Investigation of deacetylation of *N*-acetylgalactosamine and *N*-acetylglucosamine under the same acid hydrolysis conditions used for EPS resulted in rates of 100% deacetylation for GalNAc and 98% for GlcNAc (data not shown). An unknown compound from the uronic acid group was also found in *S. acidocaldarius* EPS extracts, which was not included in the developed RP-LC-MS method (no retention time matching) and could not be identified so far.

Initial experiments indicated the influence of growth substrates on *S. acidocaldarius* MW001 biofilm formation (see section “*Sulfolobus acidocaldarius* biofilm formation in different growth media”; Supplementary Figures 1, 2). Therefore, we also examined the EPS and monosaccharide composition in dependency of different growth substrates. Brock medium with 0.1% (*w/v*) NZA served as basal medium and was compared to medium additionally supplemented with either 0.2% (*w/v*) D-glucose D-xylose, or maltose. Our data indicate that the ratios of EPS components (i.e., carbohydrate, protein and eDNA) change depending on the presence of different sugars in the growth medium (Supplementary Figure 6 and Supplementary Table 4). Highest amounts of carbohydrates were detected upon the addition of D-glucose to the medium. In general, the same sugar monomer species were identified in biofilm EPS samples of all tested cultivation conditions, while the molar ratios of single constituents changed slightly depending on the added carbohydrate substrates (Supplementary Figure 7 and Supplementary Table 5).

### Reduction of RNA, DNA, and protein amounts in EPS fractions by enzymatic treatments

A relatively high proportion of ribose (26%) was detected in the hydrolyzed EPS sample of *S. acidocaldarius* MW001 biofilms (Figure 4), raising the question of its origin. In general, next to eDNA also eRNA is regarded as relevant structural nucleic acid in microbial EPS matrices (Karygianni et al., 2020). To examine whether the detected ribose moieties derived from eRNA molecules, TEM samples were subjected to RNaseA treatment. Afterward, samples were dialyzed to remove low-molecular-weight ribose molecules (MWCO 3.5 kDa and 12–14 kDa). No change in the proportion of ribose to the monosaccharide composition was found after RNaseA treatment in comparison to no treatment under the tested conditions (data not shown). Furthermore, proteins are a major compound of the *S. acidocaldarius* EPS matrix (Figure 2; Jachlewski et al., 2015). Many extracellular proteins of *S. acidocaldarius*, for example FlaB (subunit of the archaellum filament) and S-layer proteins SlaA and SlaB are known to be glycosylated (hexasaccharide composed of Glc_1_Man_2_GlcNAc_2_ plus 6-sulfoquinovose) (Peyfoon et al., 2010; Guan et al., 2016). To exclude the influence of glycosylated EPS proteins on *S. acidocaldarius* PS analyses, proteins were digested by proteinase K and the EPS protein-free sample was analyzed by RP-LC-MS (Supplementary Figure 8). Proteinase K treated EPS samples of *S. acidocaldarius* showed the same carbohydrate concentrations and monosaccharide compositions as non-treated samples. Thus, glycolsylated EPS proteins had no impact on the applied PS composition analysis.

### Determination of the exopolysaccharide size

**FIGURE 5:**
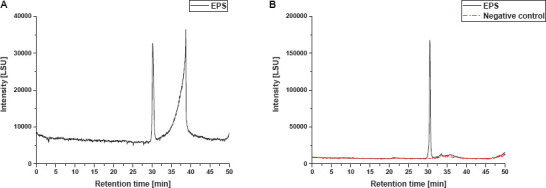
SEC-ELSD size distribution profiles of EPS fractions from *S. acidocaldarius* MW001 biofilms. **(A)** Non-treated EPS extract and **(B)** enzymatically treated EPS extract and corresponding negative control sample are shown. Both EPS sample and negative control were subjected to RNaseA, DNaseI, and proteinase K treatment for the removal of macromolecular interferences.

In order to determine the PS size an enzymatic pretreatment of the EPS fraction with RNaseA, followed bv DNaseI and proteinase K, for the degradation of eRNA, eDNA and proteins, respectively, was established to avoid interference of other polymers. RP-LC-MS analyses confirmed no changes in monosaccharide identity and composition due to the enzymatic treatments of EPS samples (data not shown). Non-treated and enzymatically treated EPS fractions (RNaseA, DNaseI and proteinase K) were compared by size exclusion chromatography (Figure 5). As carbohydrate reference for size determination a set of pullulan standards was used (Supplementary Table 6).

Chromatographic profiles obtained from EPS extracts in the non-treated sample displayed one peak at a retention time of approximately 30 min and subsequent one additional strongly fronting signal between 31 and 40 min (Figure 5A). The chromatogram of the enzymatically treated sample (RNaseA, DNaseI, proteinase K) shows only one peak with a narrow size distribution (Figure 5B, black). Thus, it appears plausible to suppose that the second signal in the non-treated sample resulted from macromolecular interference, such as proteins or eDNA, and shows a strong fronting behavior due to the non-optimized SEC method for this particular substance class. Therefore, enzymatic treatment was crucial for the size determination of the PS fraction by SEC. A distinct PS peak occurred between 29.6 to 31.4 min retention time, with the peak apex at 30.5 min, which corresponds to an elution volume of 15.3 mL and a molecular mass of ∼44 kDa for the exemplary biological replicate shown in Figure 5B. Hence, the mean PS fraction of this replicate comprised approx. 287 monosaccharide residues (with regard to the molar fraction of the different identified monosaccharides). Furthermore, four additional biological replicates of *S. acidocaldarius* MW001 biofilm PS samples were analyzed by SEC and showed a biological variance in the size of the excreted PS, ranging between ∼44 and ∼92 kDa (approx. 290—600 monosaccharide residues), resulting in a mean of approximately 71 kDa (approx. 460 monosaccharides; *n* = 5). For all replicates, only a single narrowly defined size fraction of PS was found in the SEC chromatograms.

In order to exclude an effect of the enzymatic pretreatment on PS size determination, a medium/buffer control was equivalently enzymatically treated and analyzed by SEC (negative control, Figure 5B, red). Since the samples were treated with different enzymes to remove macromolecular interferences, the used enzymes themselves and their molecular weights had to be taken into account. According to manufacturer’s information DNAseI, proteinase K and RNAseA have molecular weights of 30, 28.9, and 13.7 kDa, respectively. Thus, the mass fraction found in SEC analysis does not coincide with any molecular mass of the enzymes used; in correspondence the equally enzymatically treated negative control did not show any relevant signal in SEC analysis (Figure 5B, red).

## Discussion

Here, we report the development of a workflow for the generation of high biofilm mass, EPS isolation and chemical analyses of the monomeric composition and size of the exopolysaccharide from EPS produced by *S. acidocaldarius* biofilms.

Compared to other published work on *S. acidocaldarius* biofilms, using MTPs or dishes for cultivation (Koerdt et al., 2010; Benninghoff et al., 2017), an alternative cultivation method was established to generate high amounts of biomass necessary for subsequent EPS analysis. Different polymeric membranes were tested for their suitability to form homogeneous, thick biofilms. Cellulose-based membranes were less suitable for biofilm generation and the usage of a polycarbonate membrane resulted in the formation of visually observed macrocolonies, rather than a homogeneous biofilm. Highest yields of biofilm (5.25 mg/cm^2^) were generated with the fibrous PTFE polymer membrane, bound to a polyethylene support (Omnipore membrane, Merck), yielding a reproducibly homogeneous confluent biofilm. Compared to the cultivation of submersed biofilm at the bottom of petri dishes, a 48-fold amount of biofilm wet weight was harvested from membranes (0.11 mg/cm^2^ according to Benninghoff et al., 2021). Similar to our study, also bacterial biofilm attachment and maturation was reported to increase on fibrous membranes, such as PTFE membranes (Waheed et al., 2022).

In general, *S. acidocaldarius* MW001 biofilm amounts were increased by the addition of sugars to the growth medium, most significantly by the addition of glucose (in combination with NZA, after 4 days of cultivation in 24-well plates). In agreement with the increased biofilm amount in the presence of glucose, highest carbohydrate amounts were detected in *S. acidocaldarius* EPS fractions under this condition. Hence, the addition of glucose was beneficial to generate plenty of EPS for further PS analyses. Similar correlations between the presence of glucose in the growth medium and the production of PS or EPS were also reported for *Haloferax volcanii* biofilms (Fröls et al., 2012), *Ferroplasma acidiphilum* biofilms (Zhang et al., 2014), *Haloferax mediterranei* (Antón et al., 1988), and *Haloterrigena turkmenica* (Squillaci et al., 2016), respectively. Next to the obvious function of glucose as energy source, it may serve as sugar precursor for *S. acidocaldarius* PS synthesis. Glucose may either be activated and integrated into the exopolysaccharide (29% of glucose in PS) or converted to other PS sugar monomers, particularly mannose as a main constituent of *S. acidocaldarius* PS (35%). Although mannose is a primary component of both PS and protein *N*-glycosylation (Peyfoon et al., 2010), its synthesis pathway is yet unknown in *S. acidocaldarius*.

The isolation of water-soluble EPS was performed using the CER extraction method as described by Jachlewski et al. (2015). Compared to other extraction methods, the CER method is best suited for *S. acidocaldarius* biofilms with respect to EPS yield, impact on cell viability and its compatibility with subsequent (bio)chemical analyses (Jachlewski et al., 2015). The EPS fraction from *S. acidocaldarius* MW001 biofilms grown on membranes was dominated by 5.0 ± 0.5 fg/cell protein, followed by carbohydrates of 2.8 ± 0.6 fg/cell and 0.6 ± 0.1 fg/cell eDNA (0.1% (*w/v*) NZA, 0.2% (*w/v*) Glc medium). Beside the total biofilm formation, also the EPS composition and the ratios of protein, eDNA and carbohydrate amounts changed in response to the supplementation of different sugars to the growth medium.

Available information on the PS composition of archaeal biofilms is dominated by non-destructive analysis methods, using confocal laser scanning microscopy (CLSM) in combination with fluorophore-labeled lectins to visualize biofilm architecture and sugar moieties in the EPS matrix. Comprehensive fluorescence lectin-binding analyses are available for *Sulfolobus metallicus* DSM 6482^T^ (Zhang R. et al., 2015) and *Acidianus* sp. DSM 29099 (Zhang R.Y. et al., 2015), while only two fluorophore-labeled lectins, Concanavalin A (ConA) from *Canavalia ensiformis* binding to Glc or Man and Isolectin IB4 from *Griffonia simplicifolia* (GS-IB4) binding to Gal or GalNAc residues, were applied for the visualization of extracellular sugars in *S. acidocaldarius* biofilms (Henche et al., 2012; Orell et al., 2013, 2018; Recalde et al., 2021). CLSM analysis can serve as a qualitative indication for the presence of certain extracellular carbohydrates, but it cannot offer any information on the total sugar composition as well as quantitative amounts and ratios. In addition to that, unexpected lectin binding was reported for ConA, which was reported to interact with bacterial alginate composed of (1→4)-linked β-D-mannuronate and α-L-guluronate residues (Strathmann et al., 2002). A workflow including destroying biofilm integrity necessary for EPS isolation and advanced chemical analysis methods are beneficial for a full analysis of the extracellular monosaccharide composition in EPS.

Acid hydrolysis followed by high-performance anion exchange chromatography with pulsed amperometric detection (HPAEC-PAD) or gas chromatography coupled to MS (GC-MS) were used for the analysis of archaeal PS composition before (Lü et al., 2017; Zhang et al., 2019). Recently, a comparative study was performed in which different chromatographic methods for the analysis of monosaccharides from biological samples were assessed (Meyer et al., 2022). This study demonstrated that RP-LC-MS after monosaccharide derivatization is superior to other methods such as supercritical fluid chromatography (SFC), hydrophilic interaction liquid chromatography (HILIC), and GC in terms of separation performance, sensitivity and method standard deviation. Compared to the application of HPAEC, advantages in RP-LC-MS are also evident in terms of sensitivity (Anders et al., 2015; Squillaci et al., 2016). Furthermore, RP-LC-MS provides mass spectrometric information, which allows an assignment of unknown species to a substance class, e.g., hexoses, pentoses, uronic acids. RP-LC after derivatization has already been applied for the analysis of bacterial EPS (Rühmann et al., 2015; Qamar et al., 2021) and shows its strength in our study with regard to the detailed analysis of the PS composition of *S. acidocaldarius* biofilm.

Compared to Bacteria, information on the monomeric composition of archaeal PS is limited and only a few crenarchaeal and euryarchaeal examples were studied yet. Regarding Crenarchaea, studies are available for *Acidianus* sp. DSM 29099, *S. metallicus* DSM 6482^T^ (Zhang et al., 2019) and *Sulfolobus solfataricus* MT3 and MT4 biofilms (Nicolaus et al., 1993). For instance, *S. solfataricus* MT4 was reported to produce an exopolysaccharide from Glc/Man/GlcN/Gal in a ratio of 1.2:1.0:0.18:0.13 when grown with 0.3% (*w/v*) glucose, as detected by HPAEC-PAD and TLC (Nicolaus et al., 1993). Slightly different ratios of monosaccharides were identified in PS of *S. solfactarius* MT3 with Glc/Man/GlcN/Gal in a ratio of 1.2:1.0:0.77:0.73. A distinction was made between colloidal and capsular EPS for *Acidianus* sp. DSM 29099 and *S. metallicus* DSM6482^T^ biofilms (Zhang et al., 2019). Here, colloidal EPS were defined as soluble EPS fraction, present in the supernatant after EDTA extraction, while capsular EPS were bound to the microbial cell pellet, extracted and analyzed separately. Acid hydrolysis with TFA and HPAEC-PAD analysis of PS (>3.5 kDa) identified Glc and Man (74.4 and 25.6%) in colloidal EPS and GalN:Gal:GlcN:Glc:Xyl:Man [0.9:2.1:0.2:49.8:2.9:44.2 (%)] in capsular EPS of *Acidianus* sp. DSM 29099 (grown on S^0^). For *S. metallicus* DSM6482^T^ Gal:Glc:Man were detected in the colloidal EPS in ratios of 8.7:63.4:25.1 (%), and in the capsular EPS in ratios of 1.0:50.7:48.3 (%) (grown on S^0^) (Zhang et al., 2019).

In this study, pentoses (Rib), hexoses (Man, Glc), amino sugars (GalN, GlcN), deoxyhexoses (Rha), and acetylated species (GlcNAc) were identified in *S. acidocaldarius* EPS samples by RP-LC-MS analysis (Man/Glc/Rib/GlcN/Rha/GlcNAc/GalN in a ratio of 42:35:31:6:4:1:1). The molar proportions of mannose, glucose and ribose were the highest, making up around 90% of the detected monosaccharides. The identified amino sugars (GalN and GlcN) could originate from originally *N*-acetylated species that were deacetylated during PS hydrolysis. This is a well-known mechanism, which has been frequently studied and discussed in the literature (Einbu et al., 2007; Einbu and Varum, 2008; Knirel et al., 2019). Based on our data, observing deacetlyation rates of 100 and 98% for sugar standards of GalNAc and GlcNAc, respectively, we cannot exclude that the amino sugars found in *S. acidocaldarius* PS originate from *N*-acetylated species. Compared to CLSM analyses of *S. acidocaldarius* biofilms, using fluorophore-labeled lectins, several additional monosaccharides were identified in EPS by RP-LC-MS. Comparing the monomeric PS compositions published for different crenarchaeal species, it is obvious that glucose and mannose are the dominant monomer species detected in crenarcheal EPS samples, including that of *S. acidocaldarius* MW001. The occurrence of ribose in PS samples was also reported for bacterial EPS, including *Hahella chejuensis* (Lee et al., 2001), a bacterial/archaeal co-culture of *Thermotoga maritima* and *Methanococcus jannaschii* (Johnson et al., 2005) as well as archaeal EPS of *Halorubrum* sp. TBZ112 (HPAEC-PAD) (Hamidi et al., 2019) and *Methanosarcina mazeii* DSM6300 (GC-MS) (Veiga et al., 1997).

PS from bacterial biofilms are reported to be very long molecules with proposed molecular mass of approx. 10^4^–10^6^ Da (Mata et al., 2006, 2008; Sutherland, 2017; Wang et al., 2019). Molecular masses of euryarchaeal biofilm PS were also in the range of 10^4^–10^6^ Da, as determined by size-exclusion chromatography (SEC) for PS of *Halorubrum* sp. TBZ112 with 0.5 × 10^4^Da (Hamidi et al., 2019), *Thermococcus litoralis* with 4.1 × 10^4^Da (Rinker and Kelly, 1996), *Haloferax mucosum* with 7.65 × 10^4^ -1.52 × 10^5^Da (López-Ortega et al., 2020) and *Haloarcula hispanica* with 1.1 × 10^6^Da (Lü et al., 2017). For *Haloterrigena turkmenica*, three size fractions with significantly different molecular weights of 80.1, 20.6 and 3.8 × 10^4^Da were determined (Squillaci et al., 2016). A molecular mass of one PS with a mean of 7.1 × 10^4^ Da was identified by SEC for the crenarchaeal strain *S. acidocaldarius* MW001 in this study. This mass is comparable to reported euryarchaeal exopolysaccharide sizes. Combined with the results from the quantitative determination of the monomers, *S. acidocaldarius* PS consists of approx. 460 monomeric residues on average, assuming a linear PS structure. In order to determine a possible branching, further analyses, such as FT-IR and NMR spectroscopy, and GC-MS after methanolysis (Parolis et al., 1996, 1999; Paramonov et al., 1998; Li et al., 2018) may be performed.

Regarding the exopolysaccharide synthesis pathway in *S. acidocaldarius*, the presence of a membrane-associated synthesis, secretion and polymerization mechanism is likely. Here, especially the regulation of PS size seems to be a well-organized and controlled process, resulting in the secretion of biopolymers of a well-defined length. Previous studies observed the influence of a Lrs14-like transcriptional regulator on *S. acidocaldarius* biofilm formation and cell motility [Archaeal Biofilm Regulator 1 (abfR1)] (Orell et al., 2013). The corresponding protein (Saci_0446) was described as biofilm transcriptional repressor, controlling the expression of the archaellum operon, the archaeal adhesive pili operon and an additional gene cluster (*saci_1904-saci_1927*), encoding several glycosyltransferases and membrane proteins (Orell et al., 2013; van Wolferen et al., 2018). This gene cluster may encode proteins involved in *S. acidocaldarius* PS synthesis and excretion. In response to changing environmental conditions and nutrient availability, different glycosyltransferases may be involved in the synthesis of the PS, resulting in varying ratios of the monomers in dependency of nutrient availability (i.e., growth medium composition).

In summary, this study provides deeper insights into the EPS composition and dynamics of *S. acidocaldarius* MW001 biofilms in response to energy and carbon sources with a special focus on the monosaccharide composition and PS size. We established a protocol for the cultivation of *S. acidocaldarius* biofilm on a filter membrane as well as PS composition analyses and size determination. The presented method set displays the basis for further research on the function of PS in the EPS matrix as well as its synthesis and export by *S. acidocaldarius*.

## Data availability statement

The original contributions presented in this study are included in the article/Supplementary material, further inquiries can be directed to the corresponding authors.

## Author contributions

LK performed microbial, microscopic as well as biochemical assays and glycan labeling experiments and performed biofilm cultivation and EPS isolation. LK and MM performed biochemical assays for the quantification of EPS components. MM performed detailed analytical analyses, including sample preparation (acid hydrolyses), RP-LC-MS and SEC. CB, JW, OS, and BS acquired funding and supervised the study. LK and MM wrote the manuscript under supervision of CB, JW, OS, and BS. All authors contributed to the article and approved the submitted version.

## Abbreviations

2dGlc: 2-deoxyglucose
ANTS: 2-aminonaphthalene trisulfonic acid
Ara: arabinose
BF: biofilm suspension
CAV: cell acceleration voltage
CE: capillary electrophoresis
CER: cation exchange resin
CLSM: confocal laser scanning microscopy
ConA: Concanavalin A from *Canavalia ensiformis*
CV: crystal violet
Dex: dextrin
DMSO: dimethyl sulfoxide
dRib: 2-deoxyribose
dsDNA: double-stranded deoxyribonucleic acid
eDNA: extracellular DNA
ELSD: evaporative light scattering detector
EPS: extracellular polymeric substances
eRNA: extracellular RNA
ESI: electrospray ionization
FACE: fluorescence-assisted carbohydrate electrophoresis
Fuc: fucose
Gal: galactose
GalA: galacturonic acid
GalN: galactosamine
GalNAc: *N*-acetylgalactosamine
GC: gas chromatography
Glc: glucose
GlcA: glucuronic acid
GlcN: glucosamine
GlcNAc: *N*-acetylglucosamine
GPC: gel permeation chromatography
GS-IB4: Isolectin IB4 from *Griffonia simplicifolia*
HILIC: hydrophilic interaction liquid chromatography
HPAEC: high-performance anion-exchange chromatography
(HP)LC: (high-performance) liquid chromatography
LOD: limit of detection
LOQ: limit of quantification
Mal: maltose
Man: mannose
MRM: multiple reaction monitoring
MS: mass spectrometry
MTP: microtiter plates
MWCO: molecular weight cut-off
m/z: mass-to-charge ratio
NMR: nuclear magnetic resonance
NZA: N-Z -amine
OD: optical density
PAD: pulsed amperometric detection
PMP: 1-phenyl-3-methyl-5-pyrazolone
ProtK: proteinase K
PS: exopolysaccharides
PTFE: polytetrafluoroethylene
Rha: rhamnose
Rib: ribose
RP-LC: reversed-phase liquid chromatography
SDS-PAGE: sodium dodecylsulfate polyacrylamide gel electrophoresis
SEC: size exclusion chromatography
SFC: supercritical fluid chromatography
TCA: trichloroacetic acid
TEM: total extracellular material
TFA: trifluoroacetic acid
TQ: triple quadrupole
Xyl: xylose.
