# Supplementary material for: Exopolysaccharide composition and size in Sulfolobus acidocaldarius biofilms
Source: Front Microbiol. 2022 Sep 26;13:982745. doi: 10.3389/fmicb.2022.982745 (PMC9549778; doi:10.3389/fmicb.2022.982745)
Supplement: Supplementary file 1 [file Data_Sheet_1.pdf]

Supplementary information

Exopolysaccharide composition and size in *Sulfolobus acidocaldarius* biofilms

Laura Kuschmierz[1†], Martin Meyer[2,3†], Christopher Bräsen[1], Jost Wingender[4], Oliver J. Schmitz[2,3*] and Bettina Siebers[1*]

[1]Molecular Enzyme Technology and Biochemistry (MEB), Environmental Microbiology and Biotechnology (EMB), Centre for Water and Environmental Research (CWE), University of Duisburg-Essen, Essen, Germany
[2]Applied Analytical Chemistry (AAC), University of Duisburg-Essen, Essen, Germany
[3]Teaching and Research Center for Separation (TRC), University of Duisburg-Essen, Essen, Germany
[4]Aquatic Microbiology, Environmental Microbiology and Biotechnology (EMB), Centre for Water and Environmental Research (CWE), University of Duisburg-Essen, Essen, Germany
[†]The authors have contributed equally to this work and share first authorship.

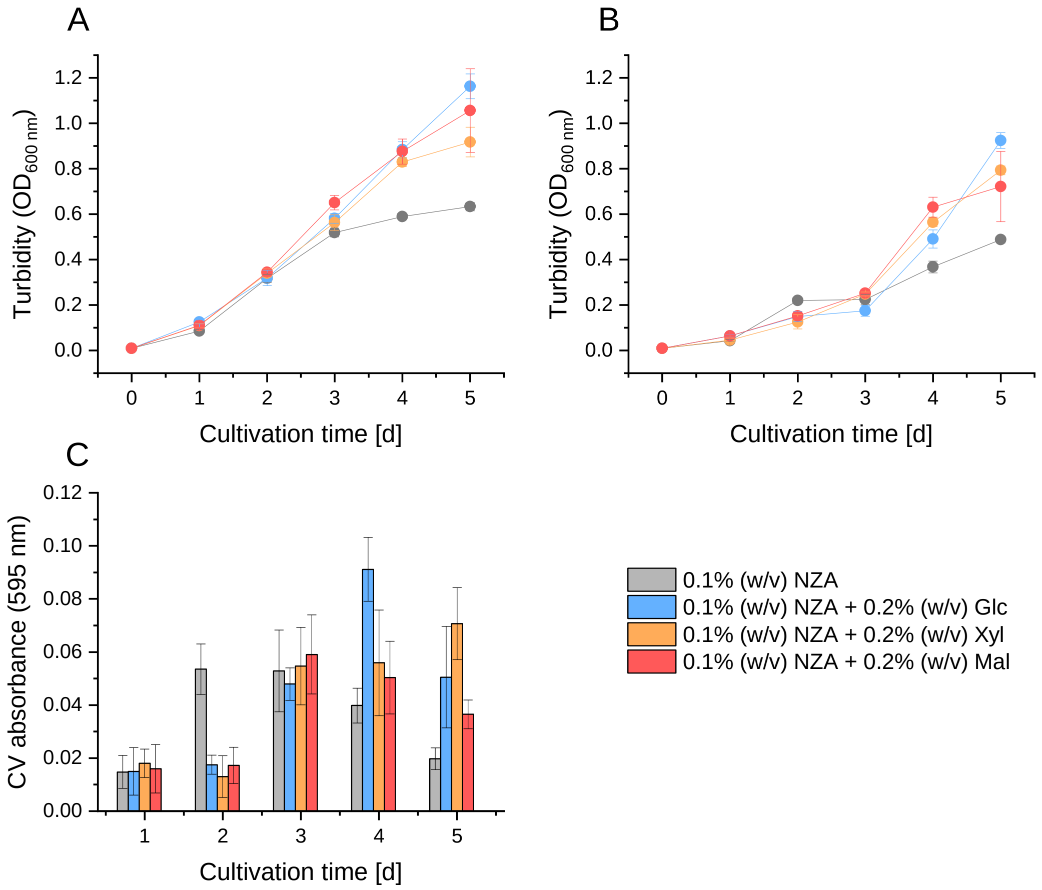

Figure S1: Effect of different growth substrates on biofilm formation of *S. acidocaldarius* MW001. Cells were statically incubated for one to five days at 76°C in 24-well microtiter plates in Brock medium (pH 3.0) containing 0.1% (*w/v*) NZA, 10 µg/mL uracil and optionally 0.2% (*w/v*) D-glucose (Glc), D-xylose (Xyl) or maltose (Mal). **A)** Total turbidity (OD_{600nm}-values) of biofilm and planktonic cells. **B)** Turbidity (OD_{600nm}-values) of planktonic cell suspensions. **C)** Quantification of biofilm biomass by crystal violet (CV) staining (n=4).

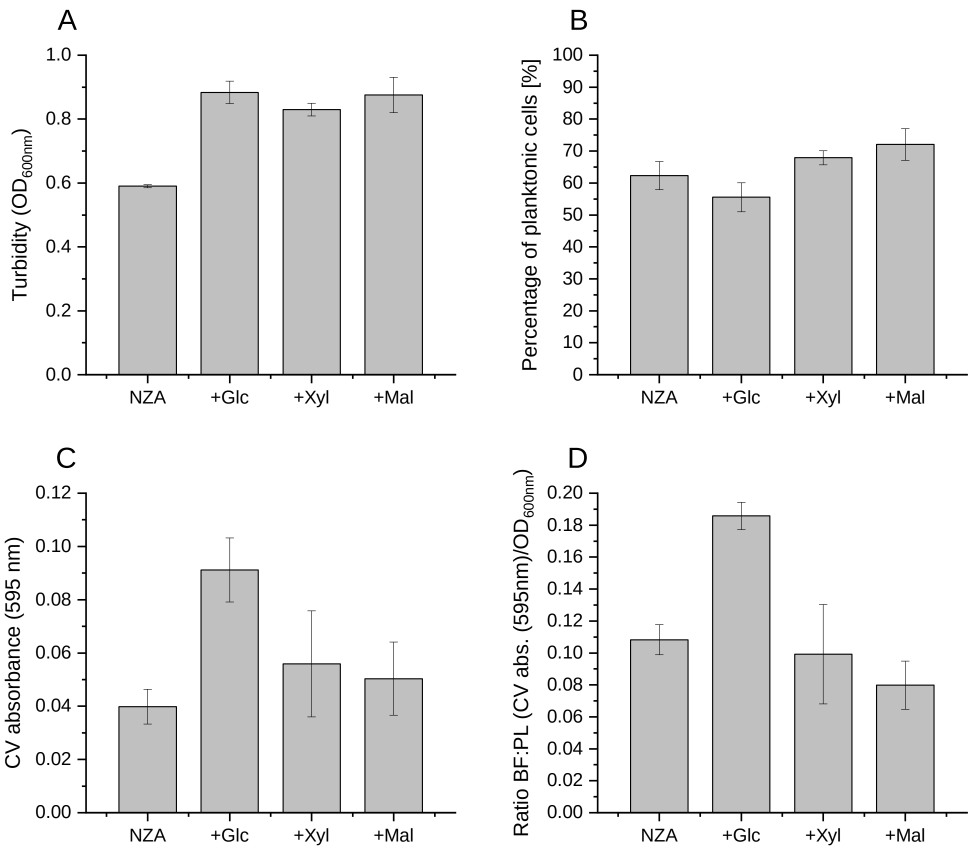

Figure S2: Effect of different growth substrates on *S. acidocaldarius* MW001 biofilm formation after 4 d of static cultivation at 76°C. Cells were incubated in 24-well microtiter plates in Brock medium (pH 3.0) containing 10 µg/mL uracil and 0.1% (*w/v*) NZA, additionally supplemented with 0.2% (*w/v*) D-glucose (Glc), D-xylose (Xyl) or maltose (Mal). **A)** Total turbidity (OD_{600nm} values) of biofilm and planktonic cells. **B)** Percentage values of planktonic cells, calculated based on the determined OD_{600nm} values of planktonic cells and total cell suspensions. **C)** Quantification of biofilm mass by crystal violet (CV) staining. Wells were washed once with Brock medium (pH 3.0) to remove planktonic cells. Firmly attached biofilm cells were quantified by CV. **D)** Correlation of CV absorbance of attached biofilm cells to turbidity values of planktonic cells (n=4).

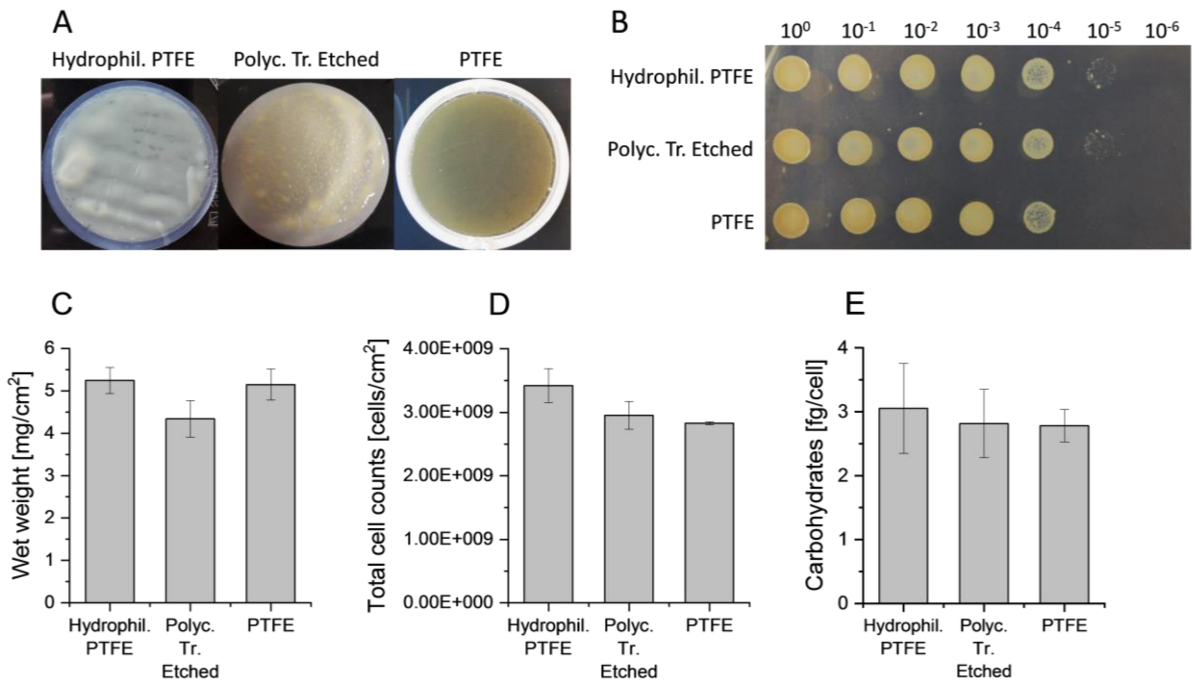

Figure S3: Comparison of three membranes with regard to *S. acidocaldarius* MW001 biofilm formation. Each membrane was inoculated with 6 * 10^5 cells and incubated floating on top of liquid Brock medium (0.1% (*w/v*) NZA, 0.2% (*w/v*) Glc, 10 µg/mL uracil) for 4 d at 76°C. **A**) Photograph of *S. acidocaldarius* biofilms on three different membranes after 4 d of static cultivation. (**B**). Spotting plates. 10 µl of undiluted culture (10^0) or diluted culture (10^{-1}-10^{-6}) were spotted on Brock-gellan gum plates (0.1% (*w/v*) NZA, 0.2% (*w/v*) Glc, 0.6% (*w/v*) gellan gum, 10 µg/mL uracil). Spot plates were incubated at 76°C for 5 d. Biofilm wet weights per area (**C**) and total cell counts (**D**) were analyzed. Carbohydrate amounts per cell were determined in EPS fractions of *S. acidocaldarius* biofilms isolated from the three different membranes (**E**). Hydrophil. PTFE: hydrophilic polytetrafluoroethylene (PTFE) membrane (Omnipore, Merck); Polyc. Tr. Etched: polycarbonate track etched filter (Sartorius); PTFE: PTFE filter (Sartorius; compare Tab. S1 for more information) (n=3).

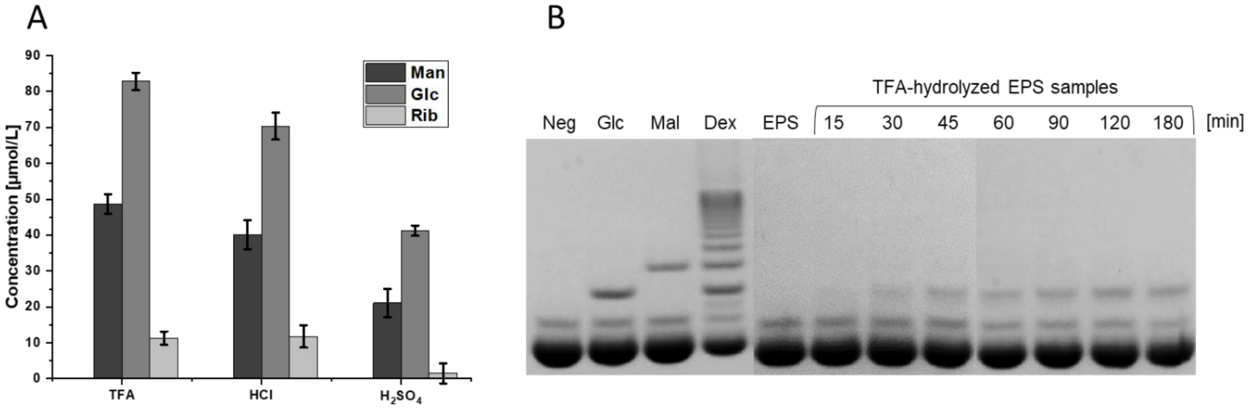

Figure S4: Evaluation of acidic hydrolyses of *S. acidocaldarius* MW001 exopolysaccharides in EPS fractions. **A**) Obtained concentrations of mannose (Man), glucose (Glc) and ribose (Rib) in EPS fractions hydrolysed with different acidic hydrolysis methods (TFA: 3 h at 95°C, neutralization with NH_4OH; HCl: 48 h at 100°C, neutralization with NaOH; H_2SO_4: 0.5 h at 0°C, 2 h at 30°C, 3 h at 100°C, neutralisation with K_2CO_3). Biofilms were cultivated with 0.1% (*w/v*) NZA, 0.2% (*w/v*) dextrin and 10 µg/mL uracil for 4 d at 76°C. **B**) Time-dependent increase in monosaccharide signals of trifluoroacetic acid (TFA)-hydrolyzed EPS samples of *S. acidocaldarius* MW001 biofilms. Hydrolysis samples were 30-fold concentrated, subjected to 2-aminonaphthalene trisulfonic acid (ANTS) labelling and visualized by fluorescence-assisted carbohydrate electrophoresis (FACE). Water was used as negative control (Neg) in ANTS labelling reactions and glucose, maltose and dextrin as mono-, di- and polysaccharide references. The ANTS labelling reaction is based on a stoichiometric addition of one label molecule per reducing end of a carbohydrate. EPS: non-hydrolyzed EPS sample.

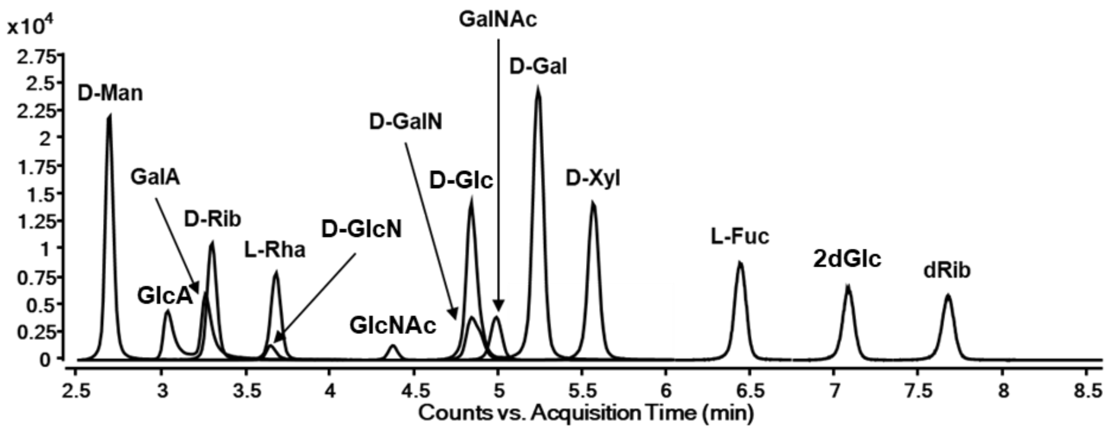

Figure S5: Separation performance of RP-LC-MS method. Overlaid multiple reaction monitoring (MRM) chromatogram for separation of 15 1-phenyl-3-methyl-5-pyrazolone (PMP)-derivatized monosaccharides from a 100 µmol/L pooled single standard sample. Separation of these 15 monomeric species was possible either by retention time or detected mass in triple quadrupole mass spectrometry (D-Man: D-mannose, GlcA: D-glucuronic acid, GalA: D-galacturonic acid, D-Rib: D-ribose, L-Rha: L-rhamnose, D-GlcN: D-glucosamine, GlcNAc: *N*-acetyl-D-glucosamine, D-GalN: D-galactosamine, D-Glc: D-glucose, GalNAc: *N*-acetyl-D-galactosamine, D-Gal: D-galactose, D-Xyl: D-xylose, L-Fuc: L-fucose, 2dGlc: 2-deoxy-D-glucose, dRib: 2-deoxy-D-ribose). Retention times and precursor masses are defined in Tab. S4.

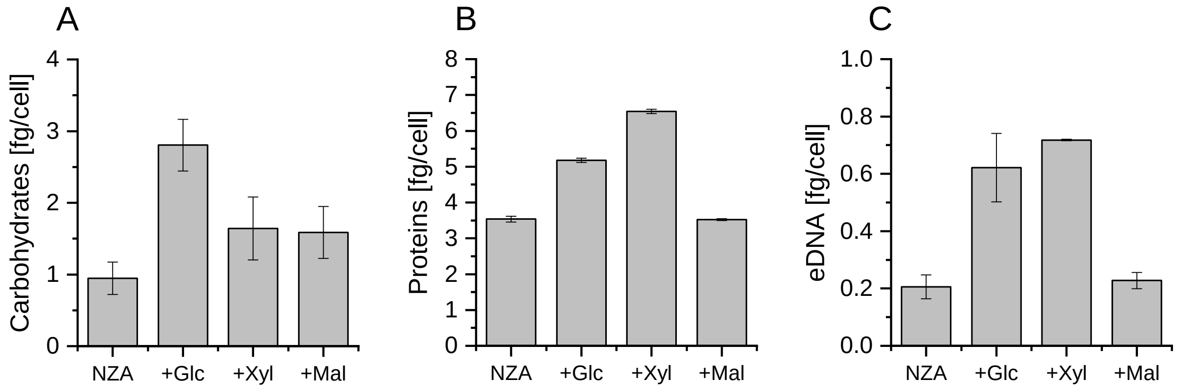

Figure S6: Influence of growth substrates on EPS composition of *S. acidocaldarius* MW001 biofilms. Cells were filtered on hydrophilic PTFE membranes and statically cultivated floating at 76°C for 4 d on liquid Brock medium (pH 3.0), containing 0.1% (*w/v*) NZA, 10 µg/mL uracil, and optionally 0.2% (*w/v*) D-glucose (Glc), D-xylose (Xyl) or maltose (Mal),. **A)** Carbohydrate amounts determined by the phenol/sulphuric acid method. **B)** Protein amounts determined by the modified Lowry assay. **C**) eDNA amounts measured using the Qubit dsDNA assay (n=3 for NZA+Glc, n=1 for NZA, NZA+Mal and NZA+Xyl; m=3; n indicates the number of biological replicates, m indicates the number of technical replicates).

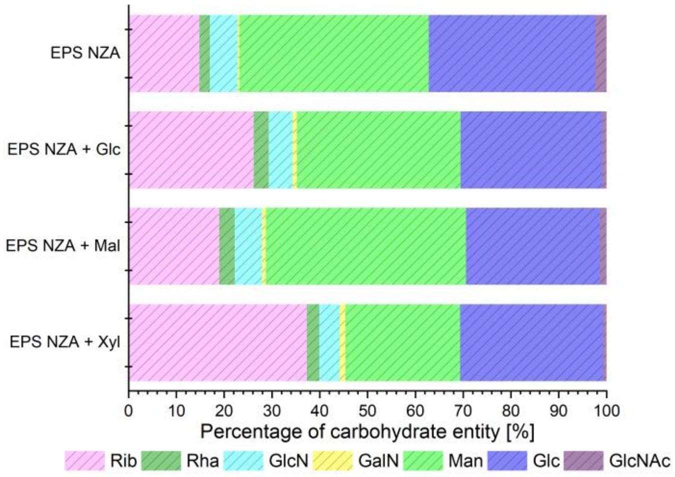

Figure S7: Monomer composition in percent of *S. acidocaldarius* MW001 exopolysaccharide in dependency of growth substrates. *S. acidocaldarius* biofilms were grown on hydrophilic PTFE membranes for 4 d at 76°C. Liquid Brock medium (pH 3.0) was supplemented with 0.1% (*w/v*) NZA, 10 µg/ml uracil and optionally 0.2% (*w/v*) D-glucose (Glc), maltose (Mal) or D-xylose (Xyl). EPS samples were generated, hydrolyzed with TFA (2 M, 95°C, 3 h) and subjected to RP-LC-MS analyses (Rib: ribose, Rha: rhamnose, GlcN: glucosamine, GalN: galactosamine, Man: mannose, Glc: glucose, GlcNAc: *N*-acetylglucosamine; n=3 for NZA+Glc, n=1 for NZA, NZA+Mal and NZA+Xyl; m=3, n indicates the number of biological replicates, m indicates the number of technical replicates). Values and standard deviations of the biological replicates are given in Table S5.

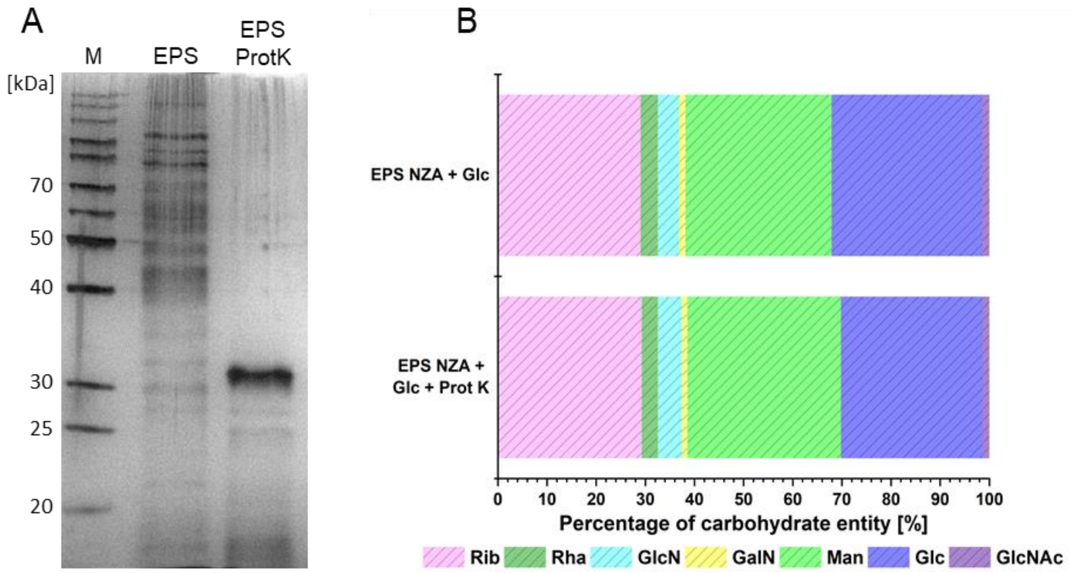

Figure S8: Protein (**A**) and exopolysaccharide (**B**) composition in EPS fractions of *S. acidocaldarius* MW001 biofilms without and with proteinase K treatment. **A**) Visualization of EPS proteins (2 µg) by SDS-PAGE and silver staining before (EPS) and after proteinase K treatment (EPS ProtK; MW of proteinase K: 28.9 kDa). Marker (M): PageRuler™ Unstained Protein Ladder (Thermo Fisher Scientific). **B**) Comparison of the exopolysaccharide composition of *S. acidocaldarius* biofilm EPS fractions without and with proteinase K treatment exemplary for one biological replicate each (Rib: ribose, Rha: rhamnose, GlcN: glucosamine, GalN: galactosamine, Man: mannose, Glc: glucose, GlcNAc: *N*-acetylglucosamine).

Table S1: Membranes tested for the cultivation of *S. acidocaldarius* MW001 biofilms. Information on membrane material, manufacturer and pore size is given. Membranes are categorized as hydrophobic (HPO) or hydrophilic (HPI). 8.9 * 10^6 *S. acidocaldarius* MW001 cells/membrane were applied. Membranes were either incubated floating on liquid Brock medium (pH 3.0), supplemented with 0.1% (*w/v*) NZA, 0.2% (*w/v*) glucose and 10 µg/mL uracil, or on solidified medium, adding 0.6% (*w/v*) gellan gum. Static cultivation was performed at 76°C for 4 d. Then, biofilms were harvested and their wet weights were determined. "n.d." (not determined) indicates either that membranes where not able to float on liquid medium, or that no cell mass could be isolated from membranes because of a lack in biofilm formation. PTFE: polytetrafluoroethylene, PE: polyethylene.

Membrane material	Manufacturer, Ref.no.	Pore size [µm]	Biofilm wet weight [mg/membrane]<br><br>Liquid medium	Biofilm wet weight [mg/membrane]<br><br>Solidified medium
Mixed cellulose ester<br>HPI	Millipore EZ-PAK, EZHAWG474	0.45	n.d.	14.2
Mixed cellulose ester<br>HPI	Pall GN-6, PA66265	0.45	24.1	14.9
Cellulose nitrate<br>HPI	Sartorius, 11407-50-ACN	0.2	n.d.	n.d.
Cellulose nitrate<br>HPI	Whatman, 7187114	0.2	n.d.	21.9
Cellulose nitrate<br>HPI	Whatman, MicroPlus, black, 10407734	0.45	n.d.	n.d.
Regenerated cellulose membrane filter, cellulose non-woven<br>HPI	Sartorius, 18406-50-N	0.45	n.d.	19.3
Polycarbonate Track Etched filter<br>HPI	Sartorius, 23006-47-N	0.4	75.3	23.6
Polyethersulfone (PES) membrane<br>HPI	Sartorius, 15406-50-N	0.45	n.d.	23.6
Hydrophilic PTFE membrane (Omnipore)<br>HPI-HPO<br>(HPI-modified; HPO PTFE bound to HPO PE support)	Merck, JHWP04700	0.45	91.0	24.7
PTFE filter<br>HPO	Sartorius, 11806-50-N	0.45	101.1	28.2

Table S2: Concentrations of mannose, glucose and ribose in *S. acidocaldarius* MW001 EPS fractions after acid hydrolysis using different methods (TFA: 3 h at 95°C, neutralization with NH_4OH; HCl: 48 h at 100°C, neutralization with NaOH; H_2SO_4: 0.5 h at 0°C, 2 h at 30°C, 3 h at 100°C, neutralisation with K_2CO_3). Biofilms were cultivated for 4 d at 76°C with 0.1% (*w/v*) NZA, 0.2% (*w/v*) dextrin and 10 µg/mL uracil.

Hydrolysis method	Concentration Mannose [µmol/L]	±	Concentration Glucose [µmol/L]	±	Concentration Ribose [µmol/L]	±
TFA	48.67	2.64	82.83	2.42	11.35	1.81
HCl	40.08	4.08	70.40	3.74	11.78	3.10
H_2SO_4	21.09	3.94	41.23	1.44	1.47	2.80

Table S3: Analytical performance of RP-LC-MS method for PMP-derivatized monosaccharides. Separation was carried out using a C18 core-shell column (Phenomenex) with an ammonia buffer-acetonitrile mobile phase gradient. Parameters for analytical performance were obtained by injection of fifteen calibration levels, limit of detection (LOD) was calculated with minimum 3x signal-to-noise ratio (S/N) and limit of quantification (LOQ) with minimum 9x S/N. Mass spectrometric detection was carried out in multiple reaction monitoring with transitions from stated precursor masses corresponding to [M-OH+2PMP]. Monosaccharide references: dRib: 2-deoxy-D-ribose, D-Rib: D-ribose, D-Xyl: D-xylose, L-Rha: L-rhamnose, L-Fuc: L-fucose, 2dGlc: 2-deoxy-D-glucose, D-GlcN: D-glucosamine, D-GalN: D-galactosamine, D-Man: D-mannose, D-Glc: D-glucose, D-Gal: D-galactose, GlcA: D-glucuronic acid, GalA: D-galacturonic acid, GlcNAc: N-acetyl-D-glucosamine, GalNAc: *N*-acetyl-D-galactosamine.

	Compound	Mass	Retention time [min]	r^2	LOD [µg/L]	LOQ [µg/L]
1	dRib	465.5	7.7	0.9999	1.3	26.8
2	D-Rib	481.5	3.3	0.9987	1.5	3.0
3	D-Xyl	481.5	5.6	0.9983	3.0	15.0
4	L-Rha	495.5	3.7	0.9989	8.2	16.4
5	L-Fuc	495.5	6.5	0.9984	8.2	16.4
6	2dGlc	495.5	7.1	0.9997	8.2	32.8
7	D-GlcN	510.5	3.7	0.9972	35.8	89.6
8	D-GalN	510.5	4.9	0.9989	9.0	35.8
9	D-Man	511.1	2.7	0.9996	3.6	9.0
10	D-Glc	511.1	4.9	0.9989	1.8	9.0
11	D-Gal	511.1	5.3	0.9992	9.0	18.0
12	GlcA	525.4	3.1	0.9905	38.8	194.1
13	GalA	525.4	3.4	0.9986	19.4	97.1
14	GlcNAc	552.5	4.4	0.9991	22.1	110.6
15	GalNAc	552.5	5.1	0.9991	22.1	44.2

Table S4: Amounts of carbohydrates, proteins and eDNA detected in EPS fractions of *S. acidocaldarius* MW001 biofilms grown on different cultivation media. The ratios of carbohydrate to protein amounts as well as carbohydrate:protein:eDNA are given (n=3 for NZA+Glc, n=1 for NZA, NZA+Mal and NZA+Xyl; m=3, n indicates the number of biological replicates, m indicates the number of technical replicates).

Growth medium	Carbohydrates [fg/cell]	Proteins [fg/cell]	eDNA [fg/cell]	Ratio carbohydrate: protein	Ratio carbohydrate: protein:eDNA
NZA	0.95 ± 0.23	3.54 ± 0.08	0.21 ± 0.04	1 : 3.7	4.5 : 17 : 1
NZA + Glc	2.80 ± 0.6	5.0 ± 0.5	0.6 ± 0.1	1 : 1.8	4.5 : 8.5 : 1
NZA + Xyl	1.64 ± 0.44	6.54 ± 0.06	0.72 ± 0.00	1 : 4	2 : 9 : 1
NZA + Mal	1.59 ± 0.36	3.52 ± 0.03	0.23 ± 0.03	1 : 2.2	7 : 15 : 1

Table S5: Molar ratios of monosaccharide composition from *S. acidocaldarius* MW001 biofilm EPS (Rib: ribose, Rha: rhamnose, GlcN: glucosamine, GalN: galactosamine, Man: mannose, Glc: glucose, GlcNAc: *N*-acetylglucosamine; n=3 for NZA+Glc, n=1 for NZA, NZA+Mal and NZA+Xyl; m=3, n indicates the number of biological replicates, m indicates the number of technical replicates).

Growth medium	Rib	Rha	GlcN	GalN	Man	Glc	GlcNAc
NZA	39	6	15	1	103	91	6
NZA + Glc	31±6	4±1	6±2	1±0	42±12	35±8	1±0
NZA + Xyl	51	4	6	2	33	41	1
NZA + Mal	23	4	7	1	51	34	2

Table S6: Calibration data for size exclusion chromatography carried out on BioSep SEC-s2000 and BioSep SEC-s4000 (Phenomenex) in series with constant elution of water at 0.5 mL min^{-1}. Pullulan Standard Set 350-700,000 (Supelco) was utilised as 10-point calibration standards with injection of 100 µL of 50 mg L^{-1} single standard solution.

Sample	Nominal mass [Da]	Log MW [Da]	Retention time [min]	Retention volume [mL]
Pullulan 342	342	2.5	46.19	23.10
Pullulan 1,030	1,030	3.0	45.11	22.55
Pullulan 6,300	6,300	3.8	40.58	20.29
Pullulan 9,800	9,800	4.0	39.01	19.51
Pullulan 22,000	22,000	4.3	35.33	17.67
Pullulan 49,400	49,400	4.7	30.75	15.37
Pullulan 110,000	110,000	5.0	25.88	12.94
Pullulan 201,000	201,000	5.3	23.57	11.78
Pullulan 334,000	334,000	5.5	22.30	11.15
Pullulan 708,000	708,000	5.9	21.40	10.70
